# Book review: Foraging with a prefrontal cortex makes all the difference

**DOI:** 10.3389/fnhum.2013.00164

**Published:** 2013-05-01

**Authors:** Rogier B. Mars, Lennart Verhagen

**Affiliations:** ^1^Department of Experimental Psychology, University of OxfordOxford, UK; ^2^Donders Institute for Brain, Cognition, and Behaviour, Radboud University NijmegenNijmegen, Netherlands

My cat is learning to catch squirrels in the back garden. It's a painfully slow process. He seems to be trying every available technique, improving step by step. He's tried sitting near the house and charging down the garden when a squirrel appeared, only for the squirrel to spot him and dash off. He's tried assaulting the squirrel when it came down the tree, only to find out that squirrels climb faster than cats. He doesn't seem to have an overview of the situation, grasping that his best strategy is to hide near the back fence, the only place where he can sneak up on the squirrel and cut off its escape route. Why isn't he capable of that seemingly simple strategic inference? A new book by Passingham and Wise (2012) provides a possible answer: because he doesn't have the large, primate prefrontal cortex (PFC).

Passingham and Wise (2012) set out to understand the organization and function of the primate PFC by combining evolutionary and ecological perspectives. Large parts of PFC, and specifically those with a granular cortical layer, are a primate invention. The authors argue that the development of these areas reflects the changes in foraging niches encountered during the evolution of the primate order.

There is a large body of work relating aspects of brain evolution to specific ecological variables, most notably foraging habits (Clutton-Brock and Harvey, 1980) and social organization (Dunbar, 1998). However, these studies have mostly focused on the relative size of the entire brain or neocortex. In contrast, work dealing with more detailed brain anatomy has tended to focus less on the evolutionary context. The unique contribution of this book is that it combines the two approaches without compromises, focusing on a detailed reconstruction of the ecological challenges encountered by the primate brain and the anatomical information that provides avenues to understanding how these challenges have been met.

## What challenges might PFC help address?

All mammals possess a neocortex, which allows them to build flexible representations of relations between environmental contingencies and reward. Within this neocortex, all mammals have some frontal cortical areas with an agranular organization, which allow them some degree of top-down control over other brain areas. Early primates moved into a novel foraging niche, that of the small branches of trees, allowing access to valuable new food sources. New adaptations such as opposable thumbs and frontally directed eyes provided an advantage in this environment. Early primates also developed additional agranular areas, including the premotor cortex allowing them to control grasping movements, and importantly also some early granular PFC areas.

When the anthropoid branch, which today houses the New and Old World Monkeys as well as the Apes, split off from the rest of the primates, their ancestors had already moved from a nocturnal to a diurnal foraging niche and increasingly depended on improved visual acuity. Early anthropoids continued this trend, and their increasing reliance on visual information was accompanied by an expansion in relative brain size, including additional visual areas. Later anthropoids became larger, requiring them to adopt other forms of locomotion, and forcing them to rely on more energy-rich food resources, such as ripe fruits and tender leaves. However, even at the best of times fruit is patchily distributed and only available for part of the year. Under these circumstances efficient foraging cannot rely on basic trial-and-error learning, it would be too slow. Therefore, anthropoids had to develop new strategies. Passingham and Wise (2012) argue that an additional set of granular prefrontal regions helped anthropoids do just that, efficiently select the most promising foraging options.

## How does it do it?

Addressing the question how PFC contributes to improved foraging, the authors first discuss the different subdivisions of PFC. They highlight that the connections of any brain area determine the information it receives and thus the function it can perform. Following this logic they place a strong emphasis on describing the differential connectivity of each part of PFC. The authors show how each region's connectivity constrains its function and, importantly, how that puts it in a unique position.

One of the prime features of PFC is the strong interconnection that exists between the different subregions. Hence, the authors next turn their attention to the role of PFC as a whole. They argue that PFC sits on top of three hierarchies: one allowing it to represent the behavioral context, one allowing it to specify goals that can be used to shape the motor plan, and one representing different outcomes along a singular dimension in terms of their current need. Each hierarchy illustrates the ability of PFC to represent and combine information processed in lower-level areas in the most abstract manner and with the highest dimensionality, whether it is the actual stimulus presented, the movement executed, or the reward delivered. By integrating these three hierarchies, PFC can map contexts to goals and predicted outcomes.

## Fast learning

What does this say about the different foraging strategies employed by my cat and me? Many animals forage successfully without a large PFC. They are capable of learning about the associations between locations and food, learn to avoid predators, and—given enough trials—can learn complex conjunctions. So what is the fundamental advance that having a PFC brings?

The problem with the non-primate way of foraging is that it is slow and error-prone. The reinforcement learning systems on which non-primates rely learn on the basis of extended feedback over many trials. In contrast, the anthropoid PFC can establish relationships between different types of information much faster and on the basis of much more abstract strategies. This is not a matter of simply adding more levels on top of an existing hierarchy; it is a matter of integrating parallel hierarchies to allow cross-inference. In that way anthropoid primates can use single events to generate goals, aiding extremely rapid learning.

Thus, granular PFC augments the ancestral learning mechanisms in a way that allows us to learn fast. Or, as Passingham and Wise put it in the subtitle of their book, PFC allows insight.
